# Lichen Simplex Chronicus Associated With Erectile Dysfunction: A Population-Based Retrospective Cohort Study

**DOI:** 10.1371/journal.pone.0128869

**Published:** 2015-06-15

**Authors:** Chao-Kuei Juan, Hsuan-Ju Chen, Jui-Lung Shen, Chia-Hung Kao

**Affiliations:** 1 Department of Dermatology, Taichung Veterans General Hospital, Taichung, Taiwan; 2 Management Office for Health Data, China Medical University Hospital, Taichung, Taiwan; 3 College of Medicine, China Medical University, Taichung, Taiwan; 4 Graduate Institute of Clinical Medical Science and School of Medicine, College of Medicine, China Medical University, Taichung, Taiwan; 5 Department of Nuclear Medicine and PET Center, China Medical University Hospital, Taichung, Taiwan; Kaohsiung Medical University HospitalKaohsiung Medical University HospitalKaohsiung Medical University Hospital, TAIWAN

## Abstract

**Background:**

An association between lichen simplex chronicus (LSC) and sexual dysfunction was explored. However, no data are available from investigations into the relationship between erectile dysfunction (ED) and LSC.

**Objectives:**

This retrospective population-based cohort study aimed to clarify the risk of ED associated with LSC.

**Methods:**

By using the Taiwan National Health Insurance Research dataset, we identified 5611 male patients who had been newly diagnosed with LSC from 2000 to 2004. The date of diagnosis was identified as the index date. LSC patients with incomplete demographic information or with a history of ED before the index date were excluded. In total, 22444 age-matched patients without LSC were randomly selected as the non-LSC group based on a 1:4 ratio. Subsequence occurrence of ED was measured until 2011. The association between LSC and the risk of developing ED was estimated using Cox proportional hazard regression model.

**Results:**

After adjusting for age and comorbidities, patients with LSC had a 1.74-fold greater risk of developing ED compared with those without LSC (95% confidence interval=1.44–2.10). LSC patients with comorbidities including diabetes, hyperlipidemia, hypertension, cardiovascular disease, peripheral arterial disease, chronic obstructive pulmonary disease, chronic kidney disease, depression, and anxiety were at a higher risk of ED compared with the non-LSC patients without comorbidities.

**Conclusions:**

LSC confers a greater risk in the development of ED. Physicians should be aware of the potential of ED occurrence in LSC patients.

## Introduction

Lichen simplex chronicus (LSC), also known as neurodermatitis, is a common chronic pruritic dermatitis characterized by lichenification of the skin caused by chronic itching and scratching. LSC affects approximately 12% of the population and occurs primarily in adults, with the highest prevalence in people aged 30–50 years [1–3]. The most common sites are the scalp, the nape of the neck, ankles, vulva, scrotum, and the extensor aspects of the extremities [1,2]. Various factors incite the development of LSC, and not all are completely understood. Psychogenic factors such as depression, anxiety, obsessive compulsive disorder, dissociative experiences, and sleep disturbances may play a crucial role, contributing to both its development and persistence [3–6]. LSC also dampens the patient’s quality of life (QOL) [7].

Erectile dysfunction (ED), otherwise known as inadequate penile erection, is defined as the inability to attain or maintain a penile erection sufficient to permit successful sexual intercourse [8]. This common disease affects primarily men older than 40 years [9,10]. The worldwide prevalence of ED is estimated to be approximately 1%–10% in men younger than 40 years, and increases with age [9,10]. In Asian populations, the prevalence rates of ED were 15.1%, 29.6%, 40.6%, 54.3%, and 70.0% for the age groups of 20–29 years, 30–39 years, 40–49 years, 50–59 years, and 60–69 years, respectively [11]. ED poses a significantly negative effect on self-esteem, interpersonal relationships, and QOL [12]. Various medical, psychological, environmental, and lifestyle factors have been suggested in the development of ED such as cardiovascular disease (CVD), diabetes mellitus (DM), hyperlipidemia, hypertension, chronic kidney disease (CKD), metabolic syndrome, and psychological distress [9–16]. Men with untreated depression and anxiety are more likely to have ED than the general population [17,18]. Thus, we postulated that an association exists between LSC and ED.

Several recent studies have attempted to address the association between LSC and sexual dysfunction [19–23]. However, all of these studies are limited in scope because they involved only a small sample, and none have explored the relationship between ED and LSC. Therefore, to identify the relationship between LSC and the subsequent risk of ED, we conducted a nationwide population-based retrospective cohort study by using a data set from the Taiwan National Health Insurance (NHI) Program. This investigation involved evaluating the incidence of ED among patients with LSC. We also examined the influence of several various comorbidities on the incidence of ED in LSC patients.

## Patients and Methods

### Data source

The NHI program was initiated in Taiwan in 1995. In 1998, the National Health Research Institutes (NHRI) established a National Health Insurance Research Database (NHIRD), released by the Bureau of National Health Insurance. To maintain individual privacy, the claims data of patients included in the NHIRD are anonymous. Comprehensive information on insured patients is included in the database, including demographic data, the dates of clinical visits, and disease diagnoses. The diagnostic codes used were based on the International Classification of Diseases, 9th Revision, Clinical Modification (ICD-9-CM).

### Study population

In this longitudinal cohort study, 5611 male patients aged 20 years and older who were newly diagnosed with LSC (ICD-9-CM 698.3), but without a previous diagnosis of ED [psychogenic ED (ICD-9-CM 302.72) or organic ED (ICD-9-CM 607.84)], were recorded in the registry of ambulatory and inpatient claims data from the 2000–2004 period. The date of the first diagnosed LSC was identified as the index date. Four study patients in the comparison cohort for every patient with LSC were randomly selected from insured people without a history of ED, frequency-matched with age (per 5 years), sex, and index-year. We thus included 5611 patients as the LSC cohort, and 22444 patients as the non-LSC cohort.

Pre-existing comorbidities comprised DM (ICD-9-CM 250.xx), hyperlipidemia (ICD-9-CM 272), hypertension (ICD-9-CM 401.xx-405.xx), CVD (ICD-9-CM 410.xx-414.xx), stroke (ICD-9-CM 430.xx-438.xx), peripheral arterial disease (PAD, ICD-9-CM 440.0x, 440.2x, 440.3x, 440.8x, 440.9x, 443.xx, 444.0x, 444.22, 444.8x, 447.8x, 447.9x), chronic obstructive pulmonary disease (COPD, ICD-9-CM 491.xx, 492.xx, and 496.xx), CKD (ICD-9-CM 580.xx-589.xx), depression (ICD-9-CM 296.2x, 296.3x, 300.4x, 311.xx), and anxiety (ICD-9-CM 300.00).

The primary outcome was the occurrence of ED, including psychogenic ED (ICD-9-CM 302.72) and organic ED (ICD-9-CM 607.84), which was determined through record linkage with the ambulatory and inpatient care of the NHIRD. All patients were observed from the index date to the diagnosed date of ED, until withdrawal from the NHI program, or the end of 2011, whichever occurred first.

### Ethics Statement

The NHIRD encrypts patient personal information to protect privacy and provides researchers with anonymous identification numbers associated with relevant claims information, including sex, date of birth, medical services received, and prescriptions. Patient consent is not required to access the NHIRD. This study was approved by the Institutional Review Board (IRB) of China Medical University (CMU-REC-101-012). The IRB specifically waived the consent requirement.

### Statistical analysis

We first compared the distributions of age (20–44, 45–59, and ≥ 60 years) and pre-existing comorbidities between the cohorts with and without LSC by using the chi-square test for categorical variables and the two-sample t-test for continuous variables. We calculated the follow-up time in person-years for each person until the diagnosis of ED, death, or the end of 2011. Crude incidence rates were calculated by dividing the number of newly diagnosed ED by the number of person-years. We analyzed the differences between groups with and without LSC during the follow-up period by using the Kaplan-Meier method with a log-rank test. The Cox proportional hazards regression model was used to assess the independent effects of LSC by adjusting for other variables. The hazard ratio (HR) was determined, along with the 95% confidence intervals (CI). Statistical significance was inferred on the basis of a two-sided *P* < 0.05. We conducted our analysis using SAS version 9.2 software (SAS Institute, Inc., Cary, North Carolina).

## Results

In our study, the LSC cohort comprised 5611 patients with LSC, and the non-LSC cohort comprised 22444 patients without LSC, with similar age distributions (Table 1). The average ages of the LSC and non-LSC cohorts were 49.46 years [standard deviation (SD) = 17.56 years] and 49.27 years (SD = 17.62 years), respectively. Patients with LSC showed a higher prevalence of DM (10.18% vs. 8.31%), hyperlipidemia (19.80% vs. 14.38%), hypertension (30.21% vs. 25.48%), CVD (17.57% vs. 13.26%), PAD (2.10% vs. 1.42%), COPD (15.68% vs. 11.34%), CKD (10.16% vs. 7.36%), depression (4.13% vs. 2.41%), and anxiety (4.21% vs. 2.55%) than those without LSC.

**Table 1:** Baseline demographic factors and comorbidity of study participants according to lichen simplex chronicus status.

	Non-LSC group N = 22444		LSC group N = 5611		p-value
Characteristics	n	%	n	%	
**Age, years**					0.99
20–44	9804	43.68	2451	43.68	
45–59	5923	26.45	1484	26.45	
≥ 60	6704	29.87	1676	29.87	
Mean (SD)^†^	49.27	(17.62)	49.46	(17.56)	0.48
**Comorbidity**					
DM	1866	8.31	571	10.18	<0.001
Hyperlipidemia	3228	14.38	1111	19.80	<0.001
Hypertension	5718	25.48	1695	30.21	<0.001
CVD	2976	13.26	986	17.57	<0.001
Stroke	722	3.22	178	3.17	0.90
PAD	318	1.42	118	2.10	<0.001
COPD	2545	11.34	880	15.68	<0.001
CKD	1651	7.36	570	10.16	<0.001
Depression	540	2.41	232	4.13	<0.001
Anxiety	573	2.55	236	4.21	<0.001

Abbreviation: LSC, lichen simplex chronicus; SD, standard deviation; DM, diabetes mellitus; CVD, cardiovascular disease; PAD, peripheral arterial disease; COPD, chronic obstructive pulmonary disease; CKD, chronic kidney disease.

^†^ Two sample t-test

**Fig 1:**
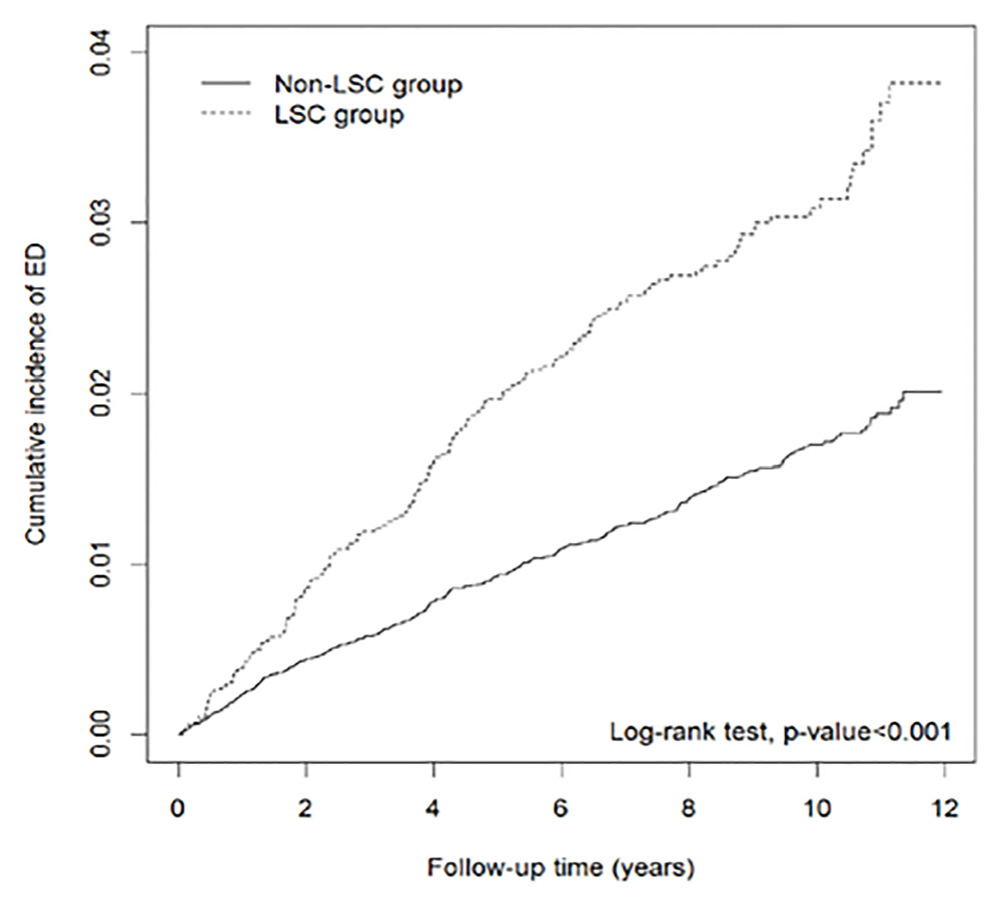
Cumulative incidence curves of ED for LSC and non-LSC groups. Abbreviation: LSC, lichen simplex chronicus; ED, erectile dysfunction.

The Kaplan-Meier survival curve indicated that patients with LSC had a significantly higher risk of developing ED (*P* < 0.001) than did the non-LSC group (Fig 1). During the follow-up period, patients with LSC had a higher incidence of ED than those without LSC (3.37 vs. 1.74 per 1000 person-years), with an adjusted HR of 1.74 (95% CI = 1.44–2.10) after adjusting for age, DM, hyperlipidemia, hypertension, CVD, stroke, PAD, COPD, CKD, depression, and anxiety (Table 2). Furthermore, the LSC group had an adjusted HR of 2.27 (95% CI = 1.20–4.30) for psychogenic ED events and 1.70 (95% CI = 1.39–2.07) for organic ED events compared with the non-LSC group. The risk of ED was significantly higher in the LSC group than in the non-LSC group in patients aged 45–59 years (adjusted HR = 1.99, 95% CI = 1.49–2.65). Regardless of the patients’ comorbidities, the LSC cohort had a higher HR of ED than did the non-LSC cohort.

**Table 2:** Incidence rates and hazard ratio for erectile dysfunction according to lichen simplex chronicus (LSC) status stratified by age and comorbidity status.

	LSC						Compared to non-LSC group			
	No			Yes			Crude HR (95% CI)		Adjusted^†^ HR (95% CI)	
Characteristics	Event no.	Person-years	IR	Event no.	Person-years	IR				
**All ED**	330	189776.47	1.74	164	48615.98	3.37	1.94	(1.61–2.34)***	1.74	(1.44–2.10)***
Psychogenic ED	24		0.13	16		0.33	2.62	(1.39–4.92)**	2.27	(1.20–4.30)*
Organic ED	306		1.61	148		3.04	1.90	(1.55–2.30)***	1.70	(1.39–2.07)***
**Age, years**										
20–44	96	87892.16	1.09	42	22491.46	1.87	1.71	(1.19–2.46)**	1.50	(1.04–2.17)*
45–59	132	52287.43	2.52	75	13093.10	5.73	2.27	(1.71–3.01)***	1.99	(1.49–2.65)***
≥ 60	102	49596.89	2.06	47	13031.41	3.61	1.77	(1.25–2.49)**	1.60	(1.13–2.27)**
**Comorbidity status** ^**‡**^										
No	132	119890.30	1.10	43	26214.15	1.64	1.49	(1.05–2.10)*	1.54	(1.09–2.17)*
Yes	198	69886.17	2.83	121	22401.83	5.40	1.91	(1.53–2.40)***	1.89	(1.51–2.37)***

Abbreviation: LSC, lichen simplex chronicus; IR, incidence rate, per 1,000 person-years; HR, hazard ratio; CI, confidence interval; ED, erectile dysfunction.

^†^ Mutually adjusted for age and comorbidity in Cox proportional hazards regression

^‡^ Patients with any one of diabetes mellitus, hyperlipidemia, hypertension, cardiovascular disease, stroke, peripheral arterial disease, chronic obstructive pulmonary disease, chronic kidney disease, depression, and anxiety were classified as the comorbidity group

* p<0.05,

** p<0.01,

*** p<0.001

Table 3 shows the combined effect of LSC and the comorbidities of DM, hyperlipidemia, hypertension, CVD, stroke, PAD, COPD, CKD, depression, and anxiety on the risk of ED related to the referent group of non-LSC and no comorbidities. We observed significant HRs of ED for patients with LSC with DM, hyperlipidemia, hypertension, CVD, PAD, COPD, CKD, depression, and anxiety compared with patients without LSC and no counterpart comorbidity (adjusted HR = 2.83, 95% CI = 1.85–4.31; 3.54, 2.64–4.74; 2.52, 1.87–3.41; 3.09, 2.23–4.29; 5.85, 3.19–10.73; 3.25, 2.32–4.54; 3.22, 2.16–4.81; 4.18, 2.45–7.15; and 4.14, 2.46–6.96, respectively). No significant interactions between LSC and DM, hyperlipidemia, hypertension, CVD, stroke, PAD, COPD, CKD, depression, and anxiety were observed (all p for interaction terms >0.05).

**Table 3:** Cox Proportional Hazard Regression Analysis for the risk of erectile dysfunction-associated lichen simplex chronicus with joint effect of comorbidity.

Variable		Event no.	IR	HR^†^ (95% CI)	p for interaction
**LSC**	**DM**				0.30
No	No	280	1.59	1.00	
No	Yes	50	3.70	1.88 (1.38–2.56)***	
Yes	No	140	3.16	1.98 (1.62–2.44)***	
Yes	Yes	24	5.51	2.83 (1.85–4.31)***	
**LSC**	**Hyperlipidemia**				0.72
No	No	247	1.51	1.00	
No	Yes	83	3.18	1.82 (1.41–2.34)***	
Yes	No	107	2.72	1.81 (1.44–2.27)***	
Yes	Yes	57	6.16	3.54 (2.64–4.74)***	
**LSC**	**Hypertension**				0.74
No	No	216	1.47	1.00	
No	Yes	114	2.64	1.38 (1.07–1.78)*	
Yes	No	100	2.84	1.95 (1.54–2.47)***	
Yes	Yes	64	4.76	2.52 (1.87–3.41)***	
**LSC**	**CVD**				0.63
No	No	263	1.57	1.00	
No	Yes	64	3.05	1.52 (1.14–2.03)**	
Yes	No	117	2.85	1.83 (1.48–2.28)***	
Yes	Yes	47	6.19	3.09 (2.23–4.29)***	
**LSC**	**Stroke**				0.84
No	No	323	1.74	1.00	
No	Yes	7	1.69	0.66 (0.31–1.41)	
Yes	No	160	3.37	1.92 (1.59–2.33)***	
Yes	Yes	4	3.64	1.45 (0.54–3.90)	
**LSC**	**PAD**				0.20
No	No	321	1.71	1.00	
No	Yes	9	4.03	1.74 (0.89–3.40)	
Yes	No	153	3.20	1.86 (1.54–2.26)***	
Yes	Yes	11	13.32	5.85 (3.19–10.73)***	
**LSC**	**COPD**				0.85
No	No	270	1.57	1.00	
No	Yes	60	3.36	1.68 (1.25–2.26)***	
Yes	No	121	2.89	1.85 (1.49–2.29)***	
Yes	Yes	43	6.38	3.25 (2.32–4.54)***	
**LSC**	**CKD**				0.73
No	No	287	1.61	1.00	
No	Yes	43	3.68	1.84 (1.33–2.56)***	
Yes	No	137	3.09	1.92 (1.56–2.35)***	
Yes	Yes	27	6.35	3.22 (2.16–4.81)***	
**LSC**	**Depression**				0.92
No	No	312	1.68	1.00	
No	Yes	18	4.37	2.29 (1.42–3.69)***	
Yes	No	150	3.20	1.90 (1.56–2.31)***	
Yes	Yes	14	8.00	4.18 (2.45–7.15)***	
**LSC**	**Anxiety**				0.68
No	No	309	1.67	1.00	
No	Yes	21	4.92	2.51 (1.61–3.91)***	
Yes	No	149	3.19	1.91 (1.57–2.32)***	
Yes	Yes	15	8.01	4.14 (2.46–6.96)***	

Abbreviation: LSC, lichen simplex chronicus; IR, incidence rate, per 1,000 person-years; HR, hazard ratio; CI, confidence interval; DM, diabetes mellitus; CVD, cardiovascular disease; PAD, peripheral arterial disease; COPD, chronic obstructive pulmonary disease; CKD, chronic kidney disease.

^†^ Adjusted for age.

* p<0.05,

** p<0.01,

*** p<0.001

We observed that the HR of ED increased with the severity of LSC, from 1.53 (95% CI = 1.25–1.87) for those who attended 3 or fewer medical visits, to 3.47 (95% CI = 1.98–6.06) for those who attended 4–6 medical visits, and to 10.35 (95% CI = 6.10–17.57) for those who attended 7 or more medical visits, compared with the non-LSC cohort (*P* trend < 0.001) (Table 4).

**Table 4:** Incidence rates and hazard ratio for erectile dysfunction risk stratified by the severity of lichen simplex chronicus.

Average frequency for medical visit, per years	N	Event no.	Person-years	IR	HR^†^ (95% CI)
**Non-LSC**	22444	330	189776.47	1.74	1.00
**LSC**					
≤3	5250	136	46409.31	2.93	1.53 (1.25–1.87)***
4–6	239	13	1642.36	7.92	3.47 (1.98–6.06)***
≥ 7	122	15	564.31	26.58	10.35 (6.10–17.57)***
p for trend					<0.001

Abbreviation: LSC, lichen simplex chronicus; IR, incidence rates, per 1,000 person-years; HR, hazard ratio; CI, confidence interval.

^†^ Adjusted for age and comorbidity in Cox proportional hazards regression.

*** p<0.001.

## Discussion

The main finding of this study was that the incidence of ED was higher in the LSC cohort than in the non-LSC cohort. LSC patients with comorbidities, including DM, hyperlipidemia, hypertension, CVD, PAD, COPD, CKD, depression, and anxiety, were associated with an increased risk of ED.

The relationship between sexual dysfunction and neurodermatitis has previously been explored. Ayyar et al. found that patients with neurodermatitis had more marital disharmony and an unsatisfactory sex life compare with those without neurodermatitis [20]. Niemeier et al. compared the sexual functions of 53 patients with psoriasis, 24 patients with neurodermatitis, and 52 controls with healthy skin [22]. Questionnaires developed by Arentewicz and their own questionnaire on sexuality were used. Patients with neurodermatitis and psoriasis had a significantly impaired sexual life compared with those with healthy skin. Mercan et al. investigated sexual functions in 31 patients with LSC, 24 patients with psoriasis, and 33 control cases by using the Arizona Sexual Experience Scale. They found that patients with psoriasis and neurodermatitis experience depression and sexual dysfunction in the course of their chronic diseases, and the frequency of sexual problems was higher in patients with neurodermatitis [19]. Van Dorssen et al. reported that sexual responsiveness and satisfaction were associated with emotional complaints and self-esteem, but did not correlate with the location or extent of skin disease [23]. Bhatia et al. found that patients with neurodermatitis had significantly higher occupational and psychosexual problems, and depression [21]. However, these previous studies did not clarify the exact relationship between LSC and ED.

To the best of our knowledge, this is the first population-based report of an association between LSC and the incidence of ED. After adjusting for age and comorbidities, we found LSC patients to be 1.74 times more likely than control patients to have been subsequently diagnosed with ED during the course of their disease (adjusted HR: 1.74; 95% CI: 1.44–2.10). Subgroup analysis based on age showed that patients aged 40–59 years had the highest prevalence of ED, followed by patients aged more than 60 years. Patients with a more severe disease, which is stratified by the frequency of medical visits per year, had a higher incidence of experiencing ED.

The mechanisms behind the increased ED risk in LSC individuals are still not fully understood.

In the present study, patients with LSC had more concomitant psychiatric disease. Psychological and emotional factors are involved in numerous cases of ED alone or in combination with other organic causes. A curial psychogenic factor related to ED is anxiety [18]. Disturbances in sexual function are also common among depressed patients. Kennedy et al. reported that approximately half of untreated depressed patients had reduced sexual desire and orgasm problems [24]. Data obtained from the Massachusetts Male Aging Study demonstrated that ED was associated with depressive symptoms [17]. Because anxiety, depression, and psychological distress are both the etiologies and consequences of LSC and ED, we postulated that psychological distress from a physically disfiguring chronic skin disease, as LSC in our study, is likely to contribute to the occurrence of ED.

In our study, we also demonstrated that LSC patients had a higher prevalence of DM, hyperlipidemia, hypertension, CVD, PAD, COPD, and CKD, compared with patients without LSC (Table 1). Previous studies have found these comorbidities have an association with ED [9–16,25–31]. In patients with DM, endothelial and smooth muscle dysfunction, and end-organ damage to the neurological system contribute to the development of ED [14]. Derangements in endothelial cell-to-cell junctions might involve in the pathogenesis of hypercholesterolemia-induced ED [15,16]. Previous studies indicated that hypoxia increases ED in COPD patients [25,26]. ED may be a presentation of systemic vascular disease, such as hypertension, PAD, as well as an early indicator of CVD [27–31]. Regarding CKD, several observational studies have documented that sexual dysfunctions was highly prevalent in patients with CKD, particularly among those on dialysis [13]. In this study, we further demonstrated a combined effect of LSC and the comorbidities on the risk of ED compared with non-LSC subjects without comorbidities. Subgroup analysis showed that this joint effect exists in LSC patients with DM, hyperlipidemia, hypertension, CVD, PAD, COPD, CKD, depression, and anxiety, respectively (Table 3).

This large nationwide, population-based database from Taiwan allowed us to explore the risk for developing ED with a low possibility of selection bias. A cohort study design enabled us to directly assess the incidence of subsequently developing ED in LSC patients. In addition, a cohort study exempted us from the contamination of potential risks because all enrolled patients of the LSC group and the matched non-LSC control group had never been diagnosed with ED before the index date of LSC diagnosis.

This study has some limitations. First, the diagnoses of LSC and ED were identified using the ICD-9-CM code from the Taiwan NHIRD. Although the diagnoses were supposed to be made clinically by an individual physician using standardized diagnostic procedures, the results of the skin pathology in the case for LSC or the self-administered International Index of Erectile Dysfunction-5 questionnaire in the case for ED are unavailable. Second, personal information (eg. family history, physical activity level, and sexual activity), disease severity, environmental exposure, and laboratory data are not documented in the database. Thus, we could not analyze and control for certain ED risk factors such as diet, obesity, smoking, alcohol consumption, and limited or lack of physical exercise [9,10]. Third, discussing sexuality remains a relatively sensitive topic in Taiwan; patients with ED may not see a specialist, but choose to receive treatment privately. In our study, the status of ED was recognized from hospital visit records, but not through direct questionnaires. This may contribute to the relatively lower incidence of ED, compared with previous studies [9,10]. Therefore, certain patients in our study could possibly be experiencing from ED, but are underreported because of Taiwanese cultural taboos. Finally, statistical significance does not always correlate with clinical significance. Population-based studies alone, without detailed individual records of mentioned data, cannot directly explain the exact mechanism through which ED is associated with LSC. Additional studies are warranted to confirm the administrative database with clinical trials.

In conclusion, patients with LSC are more likely to develop ED than are patients without such a history. LSC patients with comorbidities, including DM, hyperlipidemia, hypertension, CVD, PAD, COPD, CKD, depression, and anxiety were associated with an increased risk of ED. Physicians should be aware of the association between LSC and ED, and are recommended to advise or arrange timely sexual consultations.
